# Taking the pulse: Preimplantation genetic testing for inherited cardiac conditions

**DOI:** 10.1016/j.gimo.2023.100827

**Published:** 2023-07-20

**Authors:** Caitlin M. Finn, Frans Serpa, Usman A. Tahir

**Affiliations:** 1 Department of Medicine, Division of Cardiology, Beth Israel Deaconess Medical Center, Boston, MA; 2 Harvard Medical School, Boston, MA

## Introduction

Preimplantation genetic testing for monogenic disorders (PGT-M) is an assay used to screen embryos for a single-gene disorder before transfer during an in vitro fertilization (IVF) cycle. Couples whose offspring have a high risk of inheriting a genetic condition or predisposition caused by a known pathogenic variant(s) within their family may consider this option. Some families that are undergoing IVF to treat infertility choose to add on preimplantation genetic testing, often for conditions such as aneuploidy, as part of their IVF cycle. By only transferring embryos identified as unaffected, PGT-M significantly reduces the risk of having an affected pregnancy.

Although PGT-M has been around for a few decades, its use has remained controversial since its first human application in 1990. Originally, PGT-M was developed to detect embryos with severe or lethal childhood-onset conditions. However, its use has expanded to include late-onset conditions with reduced penetrance, such as inherited cardiac conditions (ICCs) and cancer predisposition syndromes.^1^ As the development and uptake of genetic testing continues to rapidly accelerate, it is imperative for providers in the cardiovascular clinic to be knowledgeable about PGT-M to provide high-quality care. The purpose of this article is to describe the unique factors for clinicians to consider when discussing PGT-M for ICCs. Knowledge of such factors will prepare clinicians to have informed patient discussions and effectively facilitate decision making.

### Inherited cardiac conditions

In the United States, there is currently limited guidance from professional societies on when PGT-M is appropriate to offer. In 2018, the American Society for Reproductive Medicine opinioned that PGT-M is justifiable for serious adult-onset disorders with no known, burdensome, or inadequate treatments but also for less severe or lower penetrant disorders as a matter of reproductive liberty.^1^ Although ICCs are treatable, the potential need for longitudinal cardiac screening, daily medication, an implantable cardioverter-defibrillator, lifestyle modifications, and the risk for serious disease or premature sudden cardiac death (SCD) can profoundly affect relationships, professional goals, and psychological well-being. Many patients experience anxiety and stress about SCD not only for themselves but also for their children. Parental guilt about passing down a pathogenic variant is not uncommon, which drives some couples to pursue PGT-M, and siblings who test negative may experience “survivor’s guilt” or feel neglected if a gene-positive sibling receives more attention.^2^, ^3^, ^4^ The decision to undergo PGT-M is largely influenced by a patient’s lived experience of the ICC.^4^ For example, a patient who lost a family member to SCD may be more likely to undergo PGT-M compared with an individual with a more benign past experience. Overall, parents may seek PGT-M because of the unpredictable nature of ICCs to avoid medical management (such as screening) in childhood and/or to protect future generations from serious disease.

ICCs encompass a group of heterogeneous diseases, including cardiomyopathies, arrhythmic disorders, aortopathies, and lipid disorders. Typically inherited in an autosomal dominant fashion, ICCs are a major cause of SCD among young individuals. Although these disorders are associated with significant adverse health outcomes, controversy remains regarding the application of PGT-M for ICCs because of their variable expressivity and incomplete penetrance. In other words, the presence of a pathogenic variant does not predict with certainty that a person will develop disease in their lifetime (incomplete penetrance), and a range of phenotypic expression may occur among those with the same genotype (variable expressivity), even within a family.^5^ Among those with a pathogenic variant, disease expression can range from asymptomatic throughout the lifetime to heart failure and SCD. For example, in hypertrophic cardiomyopathy only 50% of gene-positive relatives express disease by the fifth decade of life.^6^ Further, hypertrophic cardiomyopathy can be managed effectively with contemporary therapy, significantly improving morbidity and mortality associated with disease.^7^ Similar disease penetrance is observed in arrhythmogenic right ventricular cardiomyopathy, with about half of gene-positive relatives satisfying the 2010 Task Force criteria by 50 years of age.^8^ Notably, the utilization of more sensitive imaging techniques, such as late gadolinium enhancement on cardiac magnetic resonance imaging, may impact our understanding of disease penetrance in arrhythmogenic right ventricular cardiomyopathy.^9^ Approximately 25% of those who harbor a long QT syndrome pathogenic variant exhibit normal-range QTc intervals.^10^ Among those who do have a prolonged QTc interval, the cumulative probability of a sudden cardiac arrest before age 40 is 15%.^10^ The risk of cardiac events because of long QT syndrome is highest before age 40 and gradually decreases over time.^10^, ^11^, ^12^

**Figure 1:**
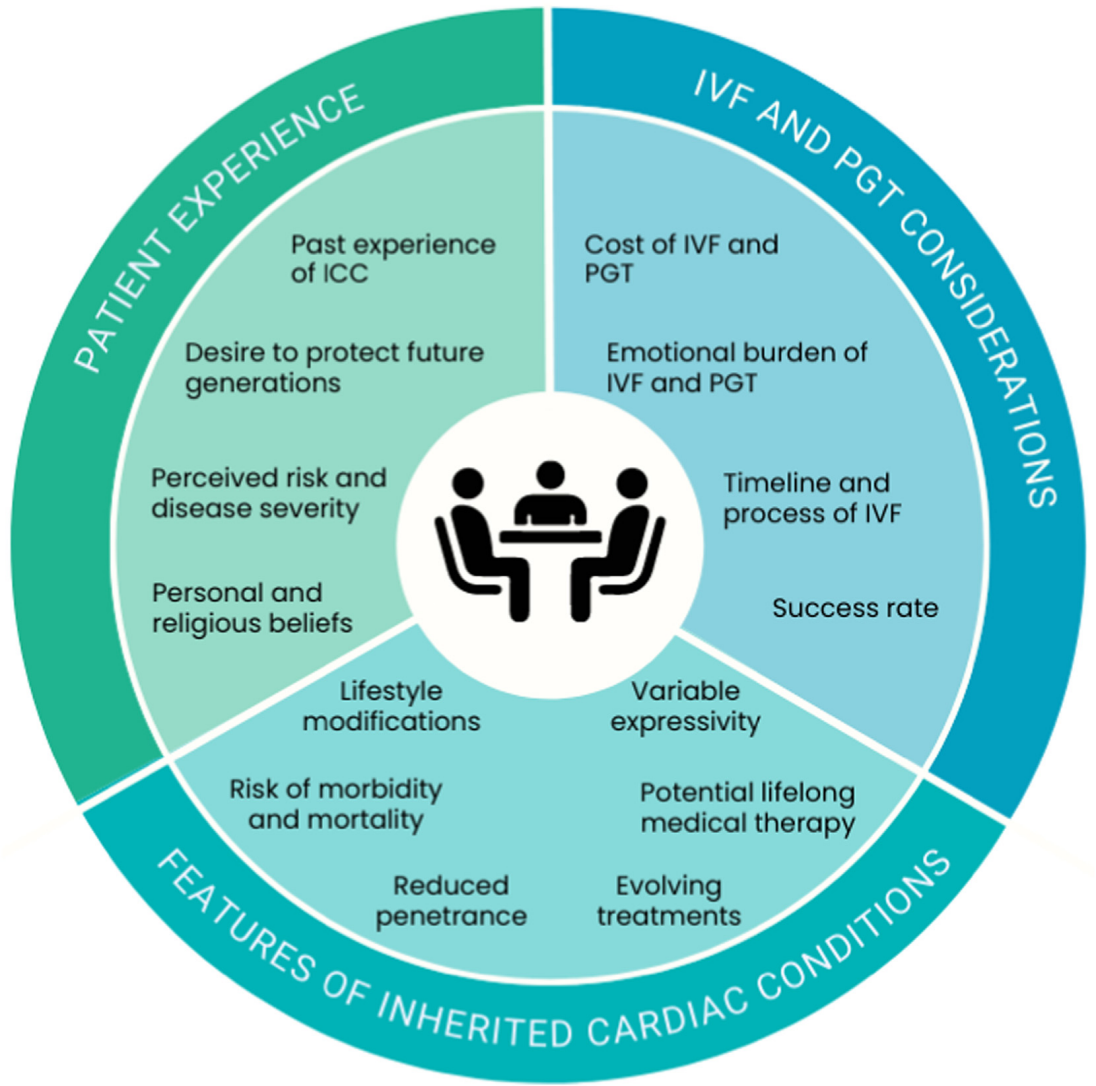
**Factors in decision making about preimplantation genetic testing for inherited cardiac conditions.** Summary of factors that influence a patient’s decision to undergo preimplantation genetic testing for inherited cardiac conditions. ICC indicates inherited cardiac condition; IVF, in vitro fertilization; PGT, preimplantation genetic testing.

Overall, clinicians should consider phenotypic heterogeneity of disease when discussing PGT-M for ICCs. It is imperative for physicians and genetic counselors to discuss all options for family planning (ie, IVF/PGT-M, conceive naturally with or without genetic testing, adoption, or IVF with donor gamete) and remain sensitive to the patient’s personal and religious beliefs. It is important to set realistic expectations when introducing IVF/PGT-M as a reproductive option by conveying this route to parentage is often time-consuming, emotionally taxing, and costly with low success rates. Additionally, it is important to note that PGT-M is considered a screening test (ie, misdiagnosis is possible but rare), and confirmatory prenatal diagnostic testing (amniocentesis or chorionic villus sampling) should be offered to confirm PGT-M results. Providers should explore the patient’s prior experience of disease and how this is shaping their decision making, while educating about phenotypic heterogeneity and IVF/PGT-M (Figure 1).

Many countries with universal healthcare systems (such as France, Italy, and the United Kingdom) have oversight for IVF and PGT-M to regulate which conditions merit coverage by government funding.^13^ Although this approach promotes equitable access, it may inadvertently affect reproductive autonomy. In the United States, most private insurance policies offer some coverage for IVF and PGT-M when a genetic condition is deemed to result in significant health problems, severe disability, or has a lethal natural history. However, coverage is subject to specific policy benefits and may result in out-of-pocket expenses. With variable insurance coverage, these procedures may have the potential to increase health disparities, disproportionately affecting people of lower socioeconomic status.^14^

Although US professional societies, such as the American Society for Reproductive Medicine, endorse the use of PGT-M for conditions that are less serious or of lower penetrance to promote reproductive autonomy, the current guidelines do not provide explicit recommendations regarding which conditions are suitable for PGT-M or a standardized process for evaluating individual cases.^1^ As a result, clinicians in the United States are responsible for deciding whether a case is ethically justifiable for PGT-M, resulting in a lack of consensus among providers. Without guidance on specific conditions from US professional societies, it is difficult to maintain a standard of care, which may exacerbate health disparities and inequitable access. There is a need for professional societies to establish best practices for PGT-M, including specific recommendations for ICCs, a systematic approach for evaluating cases, and strategies to facilitate patient decision making. Such guidelines will not only encourage more informed conversations around the use of PGT-M in clinical settings but also have the potential to provide valuable direction and coherence on a global scale.

## Conflict of Interest

The authors declare no conflicts of interest.
